# Editorial: From traditional to modern: Progress of molds and yeasts in fermented-food production, Volume II

**DOI:** 10.3389/fmicb.2022.1075162

**Published:** 2022-11-04

**Authors:** Xucong Lv, Jun Liu, Chengcheng Zhang, Van-Tuan Tran, Kap-Hoon Han, Wanping Chen

**Affiliations:** ^1^College of Biological Science and Technology, Fuzhou University, Fuzhou, China; ^2^College of Food Science and Engineering, Central South University Forestry and Technology, Changsha, China; ^3^Food Science Institute, Zhejiang Academy of Agricultural Sciences, Hangzhou, China; ^4^Department of Microbiology, University of Science, Vietnam National University, Hanoi, Vietnam; ^5^National Key Laboratory of Enzyme and Protein Technology, University of Science, Vietnam National University, Hanoi, Vietnam; ^6^Department of Pharmaceutical Engineering, Woosuk University, Wanju, South Korea; ^7^Department of Molecular Microbiology and Genetics, Georg-August-Universität Göttingen, Göttingen, Germany

**Keywords:** fermented food, mold, yeast, *Monascus*, *Saccharomyces*

Molds (filamentous fungi) and yeasts are important fermentation microorganisms that have been used for the production of foods and beverages throughout the world since ancient times (Venturini Copetti, 2019). Generally, fermented foods together with their functioning microorganisms, have strong regional characteristics (Chen, 2022). Therefore, this Research Topic was launched as a sequel to our previous topic (Chen et al., 2022), which aims to offer a collection of articles associated with different types of fermentation products and processes from different regions and provide a comparable perspective for molds and yeasts in fermented food production. The articles in this collection are introduced in the following.

*Monascus* species are important fermentation molds and are well-known for their fermentation products, red mold rice, used as a food colorant, brewing starter, and monacolin K supplement (Chen et al., 2015). This topic collects five research articles related with *Monascus* genetics and secondary metabolite synthesis. Xu, Li et al. constructed a markerless genetic modification system in *Monascus ruber* M7, in which the endogenous gene *mrpyrG* instead of the resistance marker gene was used as the screening marker. Then, the authors applied the system to delete multiple genes from *M. ruber* M7 separately or continuously without any resistance marker gene and found that the average gene replacement frequency of Δ*mrpyrG*Δ*mrlig4* was about 18 times higher than that of the wild-type. The markerless and highly efficient genetic modification system constructed in the current study will not only be used for multi-gene simultaneous modification in *Monascus* spp. and also lays a foundation for investigating the effects of multi-genes modification on *Monascus*. Ramzan et al. screened and characterized an ABC transporter involved in the transportation of phenylacetic acid in *Monascus ruber* M7. This study contributes to the clarification of the penicillin biosynthetic pathway in *Monascus*. Yin, Yang et al. investigated the effect of methionine and S-adenosylmethionine on *Monascus* pigments biosynthesis in *Monascus purpureus* RP2. The results found that the addition of methionine in fermentation significantly reduced *Monascus* pigments production by 60–70%, while the addition of S-adenosylmethionine in fermentation promoted *Monascus* pigments production by a maximum of 35%. This work also proposed a possible regulation mechanism of *Monascus* pigments biosynthesis by S-adenosylmethionine metabolism from methionine and provided a new perspective for a deep understanding of *Monascus* pigments biosynthesis regulation in *Monascus purpureus*. Yin, Zhu et al. added 20 free amino acids to the fermentation medium to evaluate their effects on *Monascus* pigments biosynthesis in *Monascus purpureus* RP2. Six amino acids, including histidine, lysine, tyrosine, phenylalanine, methionine, and cysteine, exerted significant effects on the production yield. The authors further investigated the dose-dependent and synergistic effects of these amino acids on *Monascus* pigments biosynthesis. This study would contribute to the industrial production of *Monascus* pigments. Bai et al. generated a mutant strain *Monascus purpureus* H14 with high production of water-soluble yellow *Monascus* pigments and optimized their production in submerged fermentation. The yellow pigments exhibited good tone stability when subjected to environmental factors, including pH, heat, light, and metal ions. However, their pigment values were unstable with pH, light, and high concentrations of Ca^2+^, Zn^2+^, Fe^2+^, Cu^2+^, and Mg^2+^.

This topic collects three review articles. Chi et al. summarized the principles and advantages of space microorganism breeding. Tu et al. reviewed the typical flavor substances of different types of Chinese Baijiu, the types of microorganisms involved in the brewing process, and their functions, and introduced methods that use microbial technology to enhance the flavor of Baijiu and to detect flavor substances in Baijiu. This review systematically summarizes the role and application of Chinese Baijiu flavor components and microorganisms in Baijiu brewing and provides data support for understanding Chinese Baijiu and further improving its quality. Li et al. summarized the effective approaches to producing humanized products in yeasts, and focused on yeast species selection, glycosyltransferase deletion, expression of endoglycosidase, and expression of proteins with galactosylated and or sialylated glycans. The authors also pointed out the future challenges of producing humanized glycosylated proteins.

The remaining two articles focus on the mechanism of chemical changes during fermentation. Liang et al. sequenced a *Saccharomyces cerevisiae* strain isolated from Wuyi Hongqu starters with low urea production and inferred a key gene responsible for low urea production. The results will improve our understanding on the mechanism of low urea production by *Saccharomyces cerevisiae* during Hongqu Huangjiu fermentation and may provide a way to control the urea and ethyl carbamate contents in Hongqu Huangjiu production. Xu, Miao et al. investigated the microbial succession and volatile compound dynamics of spontaneous fermentation in Xinjiang flat peach wine. The results showed that *Kazachstania, Pichia, Aspergillus, Fructobacillus, Leuconostoc*, and *Lactobacillus* were the dominant genera during the spontaneous fermentation of flat peach wine. Meanwhile, ethyl hexanoate, 3-hexen-1-yl acetate, ethyl caprate, ethyl caprylate, phenethyl acetate, ethanol, γ-decalactone, decanal, 1-hexanoic acid, and octanoic acid endued flat peach wine with a strong fruity and fatty aroma. The authors also analyzed the relationship between microorganisms and volatile chemicals. This study provides insights into the microorganisms involved in flat peach wine fermentation and could guide the production of flat peach wine with desirable characteristics.

In general, compared to the collections of the previous topic (Chen et al., 2022), the collections of this Research Topic focused more on the changes of metabolites during fermentation and tried to find out the inner mechanism. Hopefully, this topic collection will provide a reference on how to control metabolite synthesis during fermentation.

## Author contributions

All authors contributed to this work and approved it for publication.

## Conflict of interest

The authors declare that the research was conducted in the absence of any commercial or financial relationships that could be construed as a potential conflict of interest.

## Publisher's note

All claims expressed in this article are solely those of the authors and do not necessarily represent those of their affiliated organizations, or those of the publisher, the editors and the reviewers. Any product that may be evaluated in this article, or claim that may be made by its manufacturer, is not guaranteed or endorsed by the publisher.
